# Acquired zinc deficiency

**DOI:** 10.1002/jgf2.303

**Published:** 2020-02-21

**Authors:** Kotaro Kunitomo

**Affiliations:** ^1^ Department of Internal Medicine Aso Medical Center Kumamoto Japan

**Keywords:** elderly, family medicine, internal medicine, nutrition

A 66‐year‐old man with a history of diabetes mellitus experienced anorexia six months before admission. He had lost weight (52 kg → 42 kg), and his energy levels had fallen. His vital signs were unremarkable. On physical examination, he had erythema with many wet yellowish scales over the entire face, except the eyelids (Figure 1A), and slow healing decubitus. Alopecia, diarrhea, aphthous ulcer, or taste disorder was not observed.

**Figure 1:**
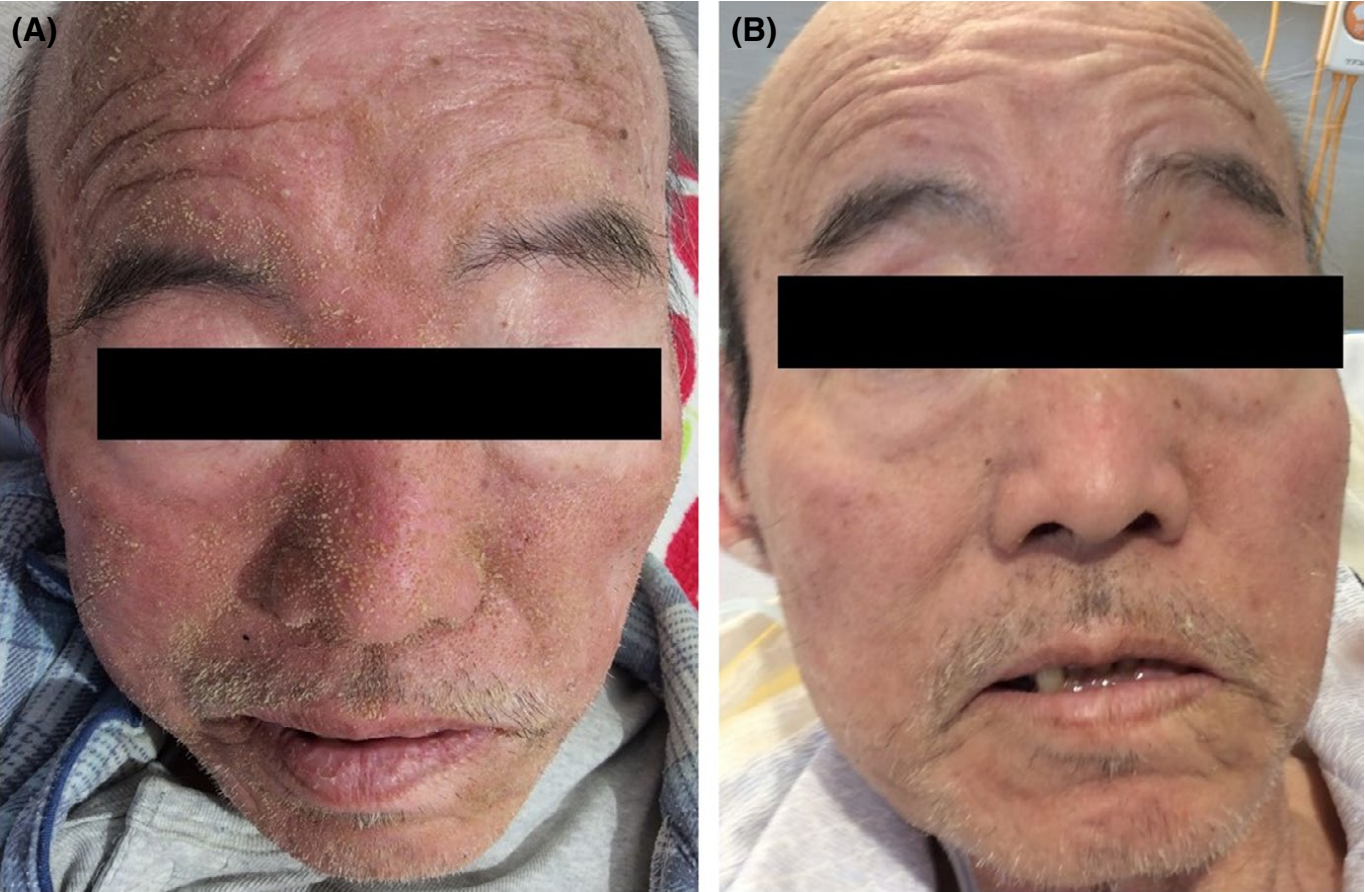
Erythema with many wet yellowish scales over the entire face, except the eyelids (A). After treatment, his facial rash improved (B)

The facial rash was considered to be seborrheic dermatitis, and ketoconazole was applied; however, there was no improvement. Laboratory testing revealed a zinc level of 35 (80‐130) μg/dL. Based on clinical and serological findings, the patient was diagnosed with acquired zinc deficiency. The patient was administered 100 mg/d of zinc acetate dihydrate, and his appetite and facial rash improved immediately (Figure 1B). After 14 days, the patient's zinc level increased to 92 μg/dL. The patient's nutritional status improved, and he was discharged. The patient's zinc intake has remained satisfactory. His vitality improved.

Dermatitis, alopecia, and diarrhea are typical symptoms of acquired zinc deficiency, in addition to impaired wound healing, dysgeusia, and night blindness. As with hereditary zinc deficiency, dermatitis of acquired zinc deficiency typically occurs in perioral and acral areas; skin findings are erythematous, scaly, and crusted plaques and erosions.^1^ However, in this case, erythema with scales was not observed in those areas, but instead over the entire face. There are few case reports of zinc deficiency with a facial rash.^2^, ^3^ Even in modern society, acquired zinc deficiency is common, occurring in approximately 17% of the world population^1^; vegetarians, alcoholics, malnourished individuals, and premature infants are most at risk.^4^ Clinicians should consider zinc deficiency as a differential diagnosis of loss of appetite with various facial rashes.

## CONFLICTS OF INTEREST

The authors have stated explicitly that there are no conflicts of interest in connection with this article.

## AUTHORS’ CONTRIBUTIONS

Kotaro Kunitomo wrote the initial draft of the manuscript.
